# The League of Mercy

**Published:** 1911-04-08

**Authors:** 


### THE LEAGUE OF MERCY.

### THE LORD MAYOR AT A MANSION HOUSE MEETING.

The Lord Mayor, with whom was the Lady Mayoress and Lady Dimsdale (Lady President), presided on March 27 at a meeting at the Mansion House in connection with the City district of the League of Mercy. There was a large attendance of lady members of the League, the occasion being a ladies' meeting. The two principal speakers were Sir Frederick Treves and Sir William Collins, M.P.

Sir Frederick Treves, having been introduced by the Lord Mayor (who welcomed the members of the League to the Mansion House), pointed out that the present age was an age of leagues political, social, patriotic, and philanthropic, but he ventured to think that there was no more noble league in the world than the League of Mercy, whose members banded themselves together for the sole purpose of carrying help, hope, and sympathy into the dark places of the earth. Those people who knew little of England were astounded at what the well-to-do classes here did for the hospitals of their country and in the interests of the poor and those who were sick and in distress. There was, indeed, no parallel anywhere of the munificence, goodwill, and splendid generosity with which the hospitals of this country were supported. If the hospitals went down a little in their capacity, the medical school would suffer at once. Their relations were so exact. They maintained the highest standard of medical efficiency among the doctors of this country, as the sole training in the great art of healing was obtained in the great hospitals which were supported by voluntary contributions. Sir Frederick Treves then went on to describe some of the wonderful treatments for diseases which the hospitals had produced, developed, and perfected. He spoke first of the Finsen Light, by the means of which lupus, one of the most loathsome diseases known to man, had been driven away. The introduction of such a cure could not have been provided privately, as it cost thousands of pounds. No doctor, however wealthy, could have done so, but the apparatus was supplied free to patients by the hospitals, which the members of the League of Mercy were so keenly supporting. Sir Frederick then referred to the *x*-ray treatment, which was now found, he said, in every cottage hospital in the country; also to the electrical treatment and the great development of antiseptic methods, while he described in touching terms the vast amount of human pain and misery that such, and other, treatment had spared to mankind. Touching upon the subject of State-aid to hospitals in this country, he expressed an opinion that the country would be far poorer by the change, that we should become poorer in sentiment and poorer in sympathy. (Applause.)

### The Voluntary Principle.

Sir William Collins, M.P., in alluding to the voluntary system as applied to hospitals, declared that it was the life and breath of such institutions. If the transfer to State-aid was ever made there would be the accompaniment of trouble and turmoil and a loss of individual effort, while it had been calculated by one statistician that such a change would add an additional 8d. in the £ on the rates of London. He was strongly opposed to anything like such a transfer, as someone had said—"when the ratepayer comes in at the door philanthropy is apt to fly out of the window." (Laughter.) The League of Mercy, which was founded by the late King Edward and in which their Majesties the King and Queen took the deepest interest, had contributed £150,000 to the King Edward Fund, or about £18,000 a year. The area of the league's operations was about to be increased, thanks to Lady Dimsdale, who had brought her energy and her influence to bear upon the work in the City district and in the organising work in connection with which she had been ably assisted by Mr. Bruce Shipway, the Hon. Secretary, and through whose efforts the present meeting had been arranged. The sum of £2,265 had been collected in the new area, thanks in no small measure to Mr. Cremerin-Joval, whose interest in hospitals was unbounded. (Applause.)

Cordial thanks were accorded to Sir Frederick Treves and Sir William Collins, the vote being proposed by Mr. F. Green and seconded by Mrs. Harrison.

### Lady Dimsdale's Speech.

Lady Dimsdale, in moving a vote of thanks to the Lord Mayor and the Lady Mayoress, said everyone present would agree with her when she said that the Lord Mayor, their President, had presided most ably, and, further, that they were all grateful to both the Lord Mayor and the Lady Mayoress for their kindness, not only in being present, but for placing the Mansion House at the disposal of the City District that afternoon. (Applause.) They all realised how few were the moments that a Lord Mayor had to give each day. There were probably few present who had not had sickness and suffering in their midst, and it was at those times that they gladly welcomed experienced and skilful nurses. While they were grateful to the doctors and surgeons for all they did for them, the nurse was always so full of kindness and tenderness to her patients and was of the greatest possible value. They should remember then that without the hospitals the nurses would never have received the adequate training they now did. That was yet another reason why they should support the hospitals. (Applause.) With regard to the shape that support should take, all sums were welcome and they would gladly receive the pennies. Lady Dimsdale then returned her thanks to the Vice-Presidents for their assistance and also to Mr. R. Bruce Shipway, the Hon. Secretary, without whose help, she thought, it would have been impossible to have held that meeting.

The Rev. R. de Courcy Laffan seconded the vote, which was carried with enthusiasm.

### The Lord Mayor on Hospitals.

The Lord Mayor, in acknowledging the compliment, said while it might be true that the Lady Mayoress did not know very much about the League of Mercy she was by no means ignorant of the work of hospitals, indeed, Lady Strong had done a very great deal for one hospital in London, and appreciated the work so highly that she was going to do a great deal more. (Applause.) With regard to what had been said concerning the amount paid during a certain period in the City, it should never be forgotten that the City of London contributed to the hospitals a larger sum than any other equal area in the British Empire. (Applause.) That being so, they should not forget that so much money given by the City for hospital work did not come through this particular agency. At the same time, the City had taken its share. (Applause.) The City area was, indeed, the most generous of all areas in the support of any object which had for its aim the betterment of the human kind. The City area was a peculiar one in every respect, and Lady Dimsdale had achieved the distinction of getting together a meeting of ladies at the Mansion

House. In spite of the unusual character of the City area, he felt that a great deal might be done in the City by the organisation of contributions through the agency of the league. That was a matter which Lady Dimsdale had already given a great deal of attention to, and how to overcome the difficulties produced by the peculiar nature of the City area. But he thought Lady Dimsdale would solve that problem as she had overcome other difficulties. With reference to State-aid or State-control for hospitals, he was strongly opposed to any suggestion of the kind, indeed, past experience in other directions justified that opposition. They had experienced in other ways the results of the transfer of control from private hands to municipal or national control, and they should all hesitate before placing anything more in such hands. It would, indeed, be a bad day if it ever came about, as it would remove personal sympathy; and it was personal sympathy that was the great and powerful factor in the administration of such institutions. It would, indeed, be a grievous mistake to ask the State to do for them what they could do for themselves.

The proceedings then terminated.

